# Host Transcriptional Response to Persistent Infection with a Live-Attenuated Porcine Reproductive and Respiratory Syndrome Virus Strain

**DOI:** 10.3390/v12080817

**Published:** 2020-07-28

**Authors:** Jayeshbhai Chaudhari, Chia-Sin Liew, Aspen M. Workman, Jean-Jack M. Riethoven, David Steffen, Sarah Sillman, Hiep L. X. Vu

**Affiliations:** 1 Nebraska Center for Virology, University of Nebraska-Lincoln, Lincoln, NE 68583, USA; jayeshvet03@gmail.com; 2 School of Veterinary Medicine and Biomedical Sciences, University of Nebraska-Lincoln, Lincoln, NE 68583, USA; dsteffen1@unl.edu (D.S.); sarah.vitosh@unl.edu (S.S.); 3 Center for Biotechnology, University of Nebraska-Lincoln, Lincoln, NE 68588, USA; chiasin.liew@unl.edu (C.-S.L.); jeanjack@unl.edu (J.-J.M.R.); 4 USDA, ARS, U.S. Meat Animal Research Center, Clay Center, NE 68933, USA; aspen.workman@usda.gov; 5 Department of Animal Science, University of Nebraska-Lincoln, Lincoln, NE 68583, USA

**Keywords:** PRRSV, persistent infection, RNA-sequencing, transcriptome, apoptosis, T-cell exhaustion

## Abstract

Both virulent and live-attenuated porcine reproductive and respiratory syndrome virus (PRRSV) strains can establish persistent infection in lymphoid tissues of pigs. To investigate the mechanisms of PRRSV persistence, we performed a transcriptional analysis of inguinal lymphoid tissue collected from pigs experimentally infected with an attenuated PRRSV strain at 46 days post infection. A total of 6404 differentially expressed genes (DEGs) were detected of which 3960 DEGs were upregulated and 2444 DEGs were downregulated. Specifically, genes involved in innate immune responses and chemokines and receptors associated with T-cell homing to lymphoid tissues were down regulated. As a result, homing of virus-specific T-cells to lymphoid tissues seems to be ineffective, evidenced by the lower frequencies of virus-specific T-cell in lymphoid tissue than in peripheral blood. Genes associated with T-cell exhaustion were upregulated. Likewise, genes involved in the anti-apoptotic pathway were upregulated. Collectively, the data suggested that the live-attenuated PRRSV strain establishes a pro-survival microenvironment in lymphoid tissue by suppressing innate immune responses, T-cell homing, and preventing cell apoptosis.

## 1. Introduction

Porcine reproductive and respiratory syndrome virus (PRRSV) is a positive sense, single stranded RNA virus that belongs to the family *Arteriviridae*, under the order *Nidovirales* [1]. Based on phylogenetic analysis, PRRSV was originally classified into two types: type 1 or PRRSV-1, which originated in Europe, and type 2 or PRRSV-2, which originated in North America. The International Committee on Taxonomy of Viruses (ICTV) recently updated the arterivirus taxonomic structure in which PRRSV-1 and PRRSV-2 are now respectively classified as two species: Betaarterivirus Suid 1 and Betaarterivirus Suid 2 [2]. PRRSV infects pigs of all ages; however, clinical manifestations are more severe when the virus infects pregnant sows and young pigs, causing reproductive failure and respiratory distress, respectively (reviewed in [3]). PRRSV is endemic in most swine producing countries worldwide, causing significant economic losses to swine producers [4].

PRRSV mainly infects cells of the monocyte/macrophage lineage [5]. CD163 is the main receptor for virus entry into susceptible cells [6]. Inside infected pigs, lung and lymphoid tissues are the main target sites of infection [7]. During acute infection, intensive inflammation is commonly observed in lung and lymphoid tissues, as there is significant infiltration with immune cells. At the same time, numerous apoptotic cells are also detected in the infected tissues during acute infection. Interestingly, the majority of apoptotic cells do not contain viral antigens, indicating that the virus induces apoptosis in bystander, non-infected cells [8]. Cytokines in the microenvironment of the infected tissues might be responsible for inducing apoptosis of bystander cells. Apoptotic cells are also detected in the endometrial-placental junctional areas of pregnant sows experimentally infected with PRRSV [9]. In vitro studies revealed that viral glycoprotein 5 (GP5) is a major inducer of apoptosis [10] although this was not reproduced in a subsequent study [11]. Besides GP5, nonstructural protein (nsp) 4 and 10 were also reported to be proapoptotic proteins [12]. Transcriptomic analysis of lung tissues of PRRSV-infected pigs during acute infection revealed a large set of differentially expressed genes (DEGs), in which increased expression of various proinflammatory cytokines (IL1A, IL1B, IL8, and IL18), chemoattractants (CCL2, CCL3, CCL4, and CCL5), and pattern recognition receptors (PRR) (TLR 3, 7, 8) were detected [13,14]. Likewise, canonical pro-apoptotic genes were upregulated during acute PRRSV infection in various tissues [15,16].

Pigs infected with PRRSV are viremic for approximately one month. After viremia resolves, the virus can establish a “smoldering” type of infection in lymphoid tissues for an extended period of time. Specifically, infectious virus can be demonstrated from tonsils of pigs experimentally infected with a wild-type PRRSV strain at 150 days post-infection (dpi) [17] while viral RNA can be detected for up to 250 dpi [18]. PRRS modified-live virus (MLV) vaccine strains can also establish persistent infection. Between 10% and 30% of pigs vaccinated with MLV vaccines carry infectious virus in their tonsil at day 60 after vaccination which can transmit the virus to naïve contact pigs [19].

Persistent infection is a common phenomenon of arteriviruses. Lactate dehydrogenase-elevating virus (LDV) (e.g., Gammaarterivirus lacdeh) and Simian hemorrhagic fever virus (SHFV) (e.g., Deltaarterivirus hemfev) establishes an asymptomatic, lifetime persistent infection in their respective natural host [20,21]. Likewise, Equine Arteritis virus (EAV) (e.g., Alphaarterivirus equid) establishes long-term persistent infection in a small portion of horses (Reviewed in [22]). Host genetics play an important role in EAV persistence. Specifically, the long-term persistence of EAV in infected horses is associated with a specific allele of the *CXCL16* gene (*CXCL16S*) [23]. Transcriptomic analysis of the ampullae, the primary site of EAV persistence in stallion, identified an enhanced expression of *CXCL16* and *CXCR6* which modulate local inflammatory responses and infiltration of lymphocytes to the site of infection [24]. Moreover, the infiltrating T-cells might be exhausted as upregulated expression of several markers of T-cell exhaustion was observed in the ampullae tissue of long-term EAV carriers.

The mechanisms of PRRSV persistence remain poorly understood. A genome-wide association study (GWAS) was conducted to identify host factors associated with PRRSV persistence. While no quantitative trait loci (QTL) with major effects on PRRSV persistence were identified, the study revealed eight genomic regions that explained at least 0.1% of the genetic variance in the GWAS [25]. These regions contain multiple genes that play a role in recruitment and activation of T-cells (T-cell transcription factor 7—TCF7), chemotactic cytokines (CCL1, CCL2, and CCL8), and T-cell migration (CD34) [25]. Recently, a study of experimental infection in pigs with PRRSV-1 revealed that during persistent infection, viral proteins are minimally expressed and viral genomes exist predominantly in the form of double stranded RNA (dsRNA) [26]. Interestingly, no significant difference in host transcriptional response between persistently infected and non-infected pigs was observed in this study. It is noteworthy that this study only analyzed a set of 189 selected swine immune genes from trachea bronchial lymph node (TBLN) tissue [26].

The goal of this study is to elucidate the putative mechanisms by which PRRSV can establish persistent infection. Genome-wide transcriptomic analysis of RNA collected from the inguinal lymph node of pigs persistently infected with a live-attenuated PRRSV strain revealed a large number of DEGs. Genes involved in innate immune response and genes encoding chemokines and receptors important for T-cell homing and trafficking were downregulated. On the other hand, genes involved in the anti-apoptotic pathway and T-cell exhaustion were upregulated. Functional studies revealed that the frequencies of virus-specific IFN-γ-secreting cells are lower in lymphoid tissue than in peripheral blood mononucleated cells (PBMCs). Collectively, the results shed important insight into the mechanisms of PRRSV persistence in the host.

## 2. Materials and Methods

### 2.1. Virus, Cells, and Reagents

The attenuated PRRSV strain designated CON90 used in this study was recovered from an infectious cDNA clone constructed using viral RNA extracted from the attenuated CON-P90 [27]. MARC-145 cells, a monkey kidney cell line [28], were cultured in Dulbecco’s modified Eagle’s medium (DMEM) supplemented with 10% fetal bovine serum (FBS) and 1 × penicillin-streptomycin (100 units/mL of penicillin, and 100 µg/mL of streptomycin (Life Technologies, Grand Island, NY, USA). PBMCs and lymph node-derived cells were cultured in complete RPMI-1640 medium (cRPMI) supplemented with 10% FBS, 1 × of GlutaMax-1 (Life Technologies, Grand Island, NY, USA), 100 units/mL of penicillin, and 100 µg/mL of streptomycin (Sigma-Aldrich, St. Louis, MO, USA). Mouse monoclonal antibody specific to PRRSV N protein SDOW17 was purchased from the National Veterinary Services Laboratories (Ames, IA, USA). Anti-porcine CD3ε (clone BB23-8E6-8C8; FITC-conjugated), anti-porcine CD4 (clone 74-12-4; PECy7 -conjugated), anti-porcine CD8 (clone 76-2-11; Alexa Flour 647 -conjugated), and anti-porcine IFN-γ (clone P2G10; PE-conjugated), were purchased from BD Biosciences (San Diego, CA, USA). Alexa Flour 488- conjugated goat anti-mouse IgG was purchased from Invitrogen (Eugen, OR, USA).

### 2.2. Animal Study and Tissue Collection

The pig experiment conducted in this study was approved by the University of Nebraska-Lincoln (UNL) Institutional Animal Care and Use Committee under the protocol number 1360. Ten 3-week-old PRRSV, porcine circovirus type 2 (PCV2), and SIV negative pigs were purchased from the Midwest Research Swine (Glencoe, MN, USA). The pigs were randomly assigned to two groups of five pigs, which were accommodated in two separate rooms in the animal biosecurity level 2 (ABSL-2) research facilities at UNL. After 1 week of acclimation, pigs in group 1 were injected with DMEM to serve as negative controls, whereas pigs in groups 2 were inoculated intramuscularly with 10^5.0^ TCID50 of live-attenuated PRRSV strain CON90. Whole blood samples with anticoagulant EDTA were collected to obtain plasma and PBMCs. Plasma samples were stored at −70 °C for measurement of viremia and antibody response. A portion of freshly isolated PBMCs were used for measurement of T cell responses while the rest of PBMCs were cryopreserved for future analysis. At 46 days post-infection (dpi), sample of inguinal lymph node (ILN) was aseptically collected from the pigs under anesthesia. One half of the INL was stored in cRPMI for lymphocyte isolation while the other half of the INL was minced and stored in TRIzol Reagent (Life Technologies, Carlsbad, CA, USA) for RNA extraction.

### 2.3. Quantification of Viral Loads

Viral loads in plasma and tissues were measured by a commercial RT-qPCR kit (Tetracore Inc., Rockville, MD, USA). Viral loads in plasma was reported as log_10_ copies per ml, whereas viral loads in tissues were reported as log_10_ copy per µg of total RNA used in the RT-qPCR reaction. For statistical purposes, samples that had no detectable levels of viral RNA were assigned a value of 0 log_10_ copies.

### 2.4. Isolation of PBMCs and ILN Cells

PBMCs were isolated from EDTA-whole blood as previously described [29,30]. Single cell suspension was isolated from ILN immediately after collection as follows. The tissue was processed to remove connecting tissue and cut into small pieces which were placed in a 70-µm nylon cell strainer (Corning, Durham, NC, USA) in the presence of cRPMI. The tissue pieces were pressed against the nylon mesh by using the plunger of a 3 mL syringe. The resulting cell suspension was collected into a 50 mL conical tube which was passed through a 70-µm cell strainer one more time to remove large tissue debris. Cells were pelleted by centrifugation at 700× *g* for 10 min at room temperature and treated with a 5 mL red blood cell (RBC) lysis buffer (Life Technologies, Carlsbad, CA, USA). RBC lysis reaction was ceased by adding ice cold PBS containing 4% FBS, followed by centrifugation at 700× *g* for 10 min at room temperature. Cell pellet was resuspended in cRPMI. To determine cell concentration and viability, samples of PBMC and ILN-derived cells were stained with acridine orange and propidium iodide (ViaStainTM AOPI Staining Solution, Nexcelom, Lawrence, MA, USA) and counted using an automatic cell counter (Cellometer Auto 2000, Nexcelom, Lawrence, MA, USA). Freshly isolated cells were used for Elispot and flow cytometric analysis. The remaining cells were cryopreserved in 10% DMSO, 40% FBS, and 50% RPMI-1640 and stored in liquid nitrogen.

### 2.5. Measurements of Immune Responses

PRRSV antibody levels in plasma were measured at the Veterinary Diagnostic Center of the University of Nebraska by using the commercial ELISA IDEXX PRRS X3 Ab Test (IDEXX Laboratories, Westbrook, ME, USA) following the manufacturer’s instructions. The serum-virus neutralization (SVN) assay was performed as previously described [31] using plasma rather than serum. Results were expressed as the log_2_ of the reciprocal of the highest dilution that showed a ≥90% reduction in the number of fluorescent foci presenting in the control wells.

The frequencies of IFN-γ-secreting cells (IFN-γ SCs) in PBMCs and ILN-derived cells were measured by using an IFN-γ Elispot assay as previously described [32,33]. Briefly two replicates of 250,000 PBMCs or ILN cells freshly collected from each pig were plated into two wells of a 96-well plate with PVDF membrane that were coated with anti-porcine IFN-γ antibody. The cells were stimulated with 100 µL of cRPMI containing 2.5 × 10^4^ TCID50 of CON90. For positive control, cells were plated at 5000 cells per well, followed by stimulation with a 100 µL cRPMI containing 10 ng/mL of phorbol 12 myristate 13-acetate (PMA) and 1 µg/mL of ionomycin. For negative control, cells were simply cultured in cRPMI. At 18 h post stimulation, the plate was washed with PBS- containing 0.05% Tween 20 (PBS-T) followed by incubation with biotin-labeled antibody against porcine IFN-γ (clone P2C11, BD Biosciences Pharmingen, San Diego, CA, USA). Spots were developed by using alkaline phosphatase-conjugated streptavidin (Southern Biotech, Birmingham, AL, USA) in conjunction with alkaline phosphatase substrate (Vector laboratories, Burlingame, CA, USA). Spots were counted and analyzed using an AID Elispot Reader Version 7.0 (AID GmbH, Strassberg, Germany).

### 2.6. Flow Cytometric Analysis of Immune Cells

Freshly isolated PBMCs and ILN cells were ex vivo stimulated with 1 × 10^5^ TCID50 of CON90 as described above. A Cocktail solution consisting of 10 ng/mL of PMA and 1 µg/mL of ionomycin was included as a positive control while cRPMI served as a negative control. At 12 hrs post-stimulation, 100 µL of cRPMI containing 1 µg/mL of Golgi-plug brefeldin A (BD Bioscience, San Jose, CA, USA) was added and further incubated for 6 hrs. Samples were centrifuged at 500× *g* for 10 min at room temperature. Cells were resuspended in flow cytometry staining buffer (FACS buffer) (PBS with 4% FBS and 0.1% sodium azide), and stained with anti-porcine CD3ε, CD4, and CD8 monoclonal antibodies and incubated on ice for 30 min in the dark. Cells were washed thrice using FACS buffer. The cells were fixed and permeabilized with 4% paraformaldehyde and 0.1% triton X-100, respectively, followed by intracellular staining with an IFN-γ antibody. Cells were analyzed by using a Cytek DxP10 cytometer (Cytek Biosciences, Fremont, CA, USA) and acquired data were analyzed using the FlowJo software (BD Biosciences, San Jose, CA, USA) with gating based upon fluorescence minus one (FMO) control. For each sample, 100,000 events were acquired. Relevant cell population was gated on CD3^+^ prior to analyzing the CD4^+^ and CD8^+^ cells (Figure S1a). IFN-γ positive cells were counted from individual CD4^+^, CD8^+^, and CD4^+^CD8^+^ double positive (DP) cells (Figure S1b).

### 2.7. RNA Sequencing

Total RNA was isolated from ILN collected from the infected and non-infected using the TRIzol reagent according to the manufacturer’s protocol. RNA was re-purified using RNeasy Mini Kit (Qiagen, Hilden, NRW, Germany) according to the manufacturer instructions, followed by a DNase treatment using Turbo DNA-free^TM^ kit (Life Technologies, V.A. Graiciuno 8, Vilnius, Lithuania) to remove DNA contamination. Purified RNA was submitted to Novogene Bioinformatics Technology Co.Ltd for RNA sequencing.

Sequencing libraries were generated using NEBNext^®^ UltraTM RNA Library Prep Kit for Illumina^®^ (NEB, Ipswich, MA, USA) following manufacturer’s recommendations and index codes were added to attribute sequences to each sample. Briefly, mRNA was purified from 1 mg of total RNA using poly-T oligo-attached magnetic beads. Fragmentation was carried out using divalent cations under elevated temperature in NEBNext First Strand Synthesis Reaction Buffer (5X). First strand cDNA was synthesized using random hexamer primer and M- MuLV Reverse Transcriptase (RNase H). Second strand cDNA synthesis was subsequently performed using DNA Polymerase I and RNase H. Remaining overhangs were converted into blunt ends via exonuclease/polymerase activities. After adenylation of 3′ ends of DNA fragments, NEBNext Adaptor with hairpin loop structure were ligated to prepare for hybridization. In order to select cDNA fragments of preferentially 150~200 bp in length, the library fragments were purified with AMPure XP system (Beckman Coulter, Beverly, MA, USA). Then 3 µL USER enzyme (NEB, Ipswich, MA, USA) was used with size-selected, adaptor-ligated cDNA at 37 °C for 15 min followed by 5 min at 95 °C before PCR. Then PCR was performed with Phusion High-Fidelity DNA polymerase, Universal PCR primers and Index (X) Primer. Finally, PCR products were purified using AMPure XP system and library quality was assessed on the Agilent Bioanalyzer 2100 system. The libraries were sequenced on an Illumina platform Hiseq 2000 and approximately 40 million raw 125 bp/150 bp paired-end) reads were generated.

### 2.8. Differential Expression Analysis

RNA-Seq reads were first trimmed with Trim Galore version 0.6.4 [34,35], which include filtering reads shorter than 30 bp and low-quality ends from reads (Phred score: <20) and the option of removing reads with Ns. The quality of reads before and after trimming was checked with FastQC version 0.11.7 [36]. The filtered reads were aligned to the *Sus scrofa* genome (Sscrofa11.1; GCF_000003025.6) using TopHat version 2.0.14 [37,38,39] with a read mismatch of 0 and a maximum multi hits of 5 and further default parameters. Alignment files generated by TopHat were then used to generate gene counts using HTSeq version 0.9.1 (htseq-count), with a minimum alignment quality of 10 [40]. A data matrix of gene counts for all samples was created using a custom Python script and the data matrix was used to run the differential gene expression analysis in DESeq2 version 1.22.1 [41]. Genes with an adjusted *p* value smaller than 0.05 and a log_2_ fold change larger than 1 were considered as differentially expressed. The differential expression result table generated by DESeq2 was annotated with gene information obtained from Ensembl BioMart for *Sus scrofa*, supplemented by annotation from the GTF file. To visualize the level of PRRSV RNA genome, reads mapped to the PRRSV CON90 genome were counted base by base, normalized to counts per million and subsequently used to generate a coverage track using deepTools version 3.4.3 [42].

### 2.9. GO Enrichment Analysis and KEGG Pathway Analysis of Differentially Expressed Genes

Gene Ontology (GO) in the database (http://www.geneontology.org/) is an international standardized classification system for gene function, and it supplies a set of controlled vocabulary to comprehensively describe the properties of genes and gene products. GO enrichment analysis of DEGs was implemented by the GOseq R package [43], in which gene length bias was corrected. GO terms with corrected *p*-value less than 0.05 were considered significantly enriched by differentially expressed genes. Kyoto Encyclopedia of Genes and Genomes (KEGG) is a database resource for understanding high-level functions and utilities of the biological system, such as the cell, the organism, and the ecosystem, from molecular-level information, especially large-scale molecular datasets generated by genome sequencing and other high-throughput experimental technologies (http://www.genome.jp/kegg/). KOBAS version 3.0 [44] was used to test the statistical enrichment of differential expression genes in KEGG pathway.

### 2.10. Statistical Analysis

Virus-specific T-cell data were analyzed by unpaired *t*-test analysis using GraphPad prism software version 8.3.1 (GraphPad Software, LLC, San Diego, CA, USA). A *p* < 0.05 was considered significant.

### 2.11. Data Access

Sequencing data from RNA-Seq were deposited in the NCBI GEO and are available under accession number GEO: GSE153174

## 3. Results

### 3.1. The Attenuated PRRSV Strain CON90 Established Persistent Infection in Pigs

**Figure 1:**
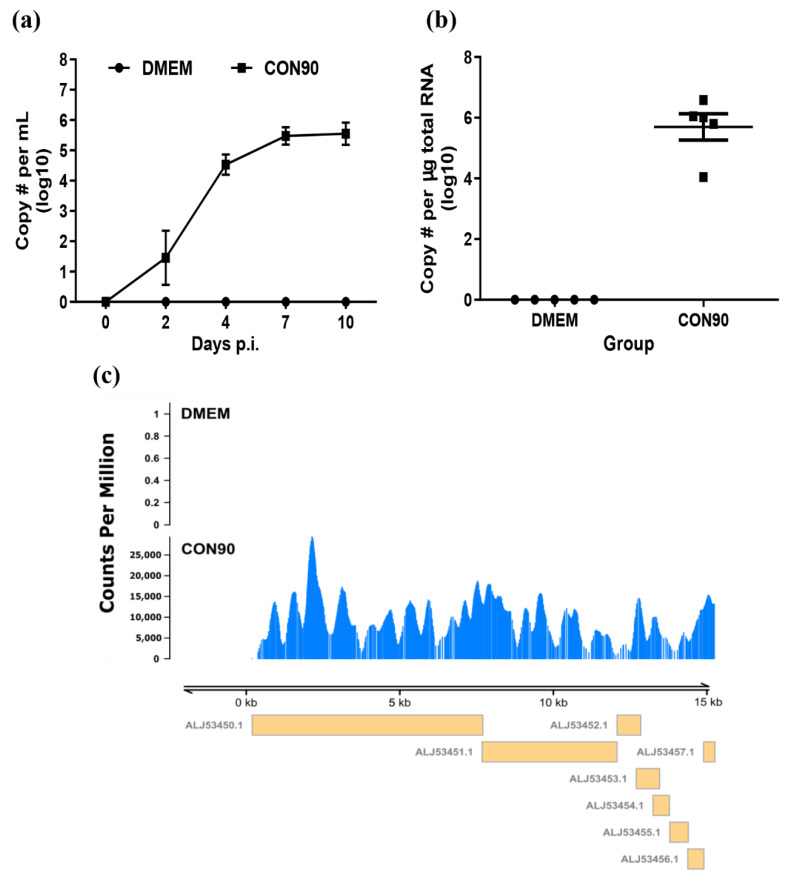
Post-inoculation viral RNA detection in plasma and inguinal lymph node (ILN) tissue. (**a**) Viral RNA in plasma at indicated dpi and (**b**) viral RNA level in ILN tissue at 46 dpi as determined by a commercial qRT-PCR kit (Tetracore Inc., Rockville, MD, USA). Dataset represents mean ± S.E.M values calculated from five pigs in each treatment group. (**c**) Coverage plot of the RNA reads mapped to CON90 genome. *Y* axis represents the count per million (cpm) while the *X* axis represents the viral genome. Yellow boxes under the plot with GenBank accession numbers indicate the viral open reading frames.

All pigs inoculated with CON90 became viremic starting from 2 dpi. The viremia level increased and peaked at 7 dpi (Figure 1a). Pigs inoculated with CON90 did not displayed any clinical signs throughout the course of this study. At 46 dpi, ILN was collected and RNA was extracted. RT-PCR analysis revealed that viral RNA genome was detected in all five CON90-infected pigs but none of the sham-inoculated pigs (Figure 1b). Subsequently, the RNA samples were subjected to RNA-seq. An average of 32 million paired-end reads were obtained for each RNA sample (Table S1). To confirm the presence of viral RNA during persistent infection in pigs, the reads were mapped to the CON90 genome. Viral RNA reads were only detected from ILN of CON90-infected pigs which mapped throughout the viral genome (Figure 1c). The results demonstrate that all five pigs in this study were persistently infected with the attenuated PRRSV strain CON90.

### 3.2. Robust Host Responses to CON90 Persistent Infection in ILN

**Figure 2:**
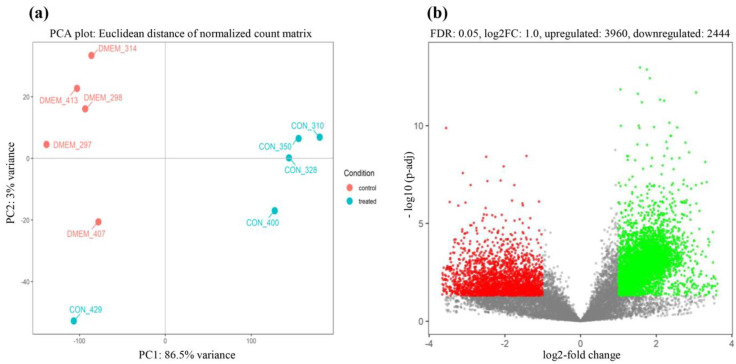
Two-dimensional principal component analysis (PCA) plot and volcano plot. (**a**) Principal component analysis (PCA) score plot. The control animals are indicated by red dots labeled “DMEM_ animal number,” while the CON90-infected animals are indicated by cyan dots labeled “CON_animal number”; (**b**) the volcano plot of differentially expressed genes between CON90-infected and control pigs. Red dots denote significantly down-regulated genes (*p* < 0.05, log_2_ fold change ≤−1), green dots indicated significantly up-regulated genes (*p* < 0.05, log_2_ fold change ≥1), and grey dots represent genes that were not differentially expressed.

To examine the host responses to CON90 persistent infection, RNA reads were mapped to the reference pig genome (Sscrofa11.1; GCF_000003025.6). Principal component analysis (PCA) indicated that control and CON90-infected pigs formed separated clusters indicating that they had distinct transcriptional profiles (Figure 2a). The infected pigs showed more variability than the control pigs. In addition, one pig in the CON90-infected group (CON_429) showed a unique transcriptional profile, as it associated closer to the control pigs. This association is likely due to the different genetic makeup of this pig than the other four animals within this group.

Direct comparison of RNA transcripts between control and CON90-infected animals revealed a profound change in the transcriptional profiles in the ILN tissue of CON90-infected pigs. Out of 17553 genes in the annotated porcine genome, there were 6404 DEGs (FDR < 5%, |log_2_| fold change ≥ 1), of which 3960 DEGs were upregulated and 2444 DEGs were downregulated (Figure 2b and Table S2).

**Figure 3:**
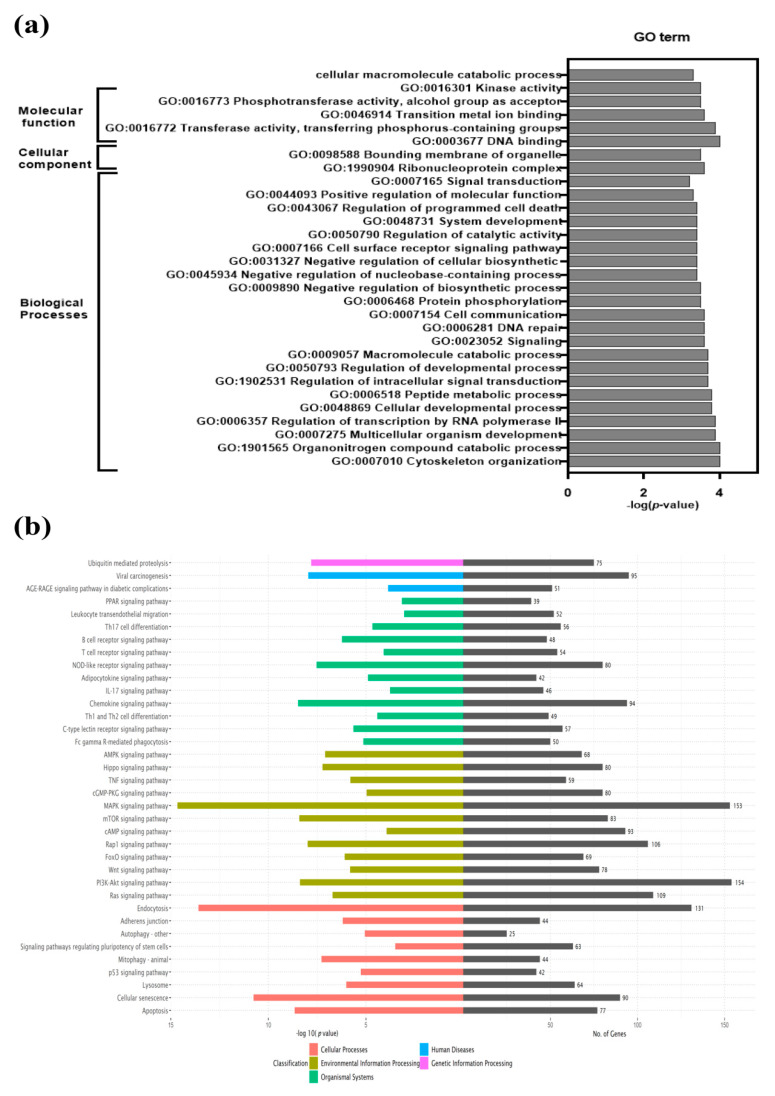
Gene Ontology (GO) enrichment and Kyoto Encyclopedia of Genes and Genomes (KEGG) pathway analysis of differentially expressed genes (DEGs). (**a**) GO analysis of DEGs at 46 dpi. Top 30 most significant GO categories *p*-value < 0.05 are shown; (**b**) KEGG pathway enrichment analysis was performed for DEGs. The graph depicts the top 36 overrepresented KEGG pathways. Pathways showing, *p*-value < 0.05 are considered statistically significantly overrepresented. The left side indicates the enrichment score (*p*-value) while the right side of the graph indicates the number of genes in the respective pathways.

To understand their biological functions, DEGs were subjected to GO enrichment analysis. GO terms having a corrected value of *p* < 0.05 were considered statistically significant (Table S3). Significantly enriched GO terms were grouped under three categories: biological processes, molecular function, and cellular components. Thirty highly enriched GO terms in CON90-infected pigs are shown (Figure 3a). A large number of DEGs are enriched into the category of biological processes (cytoskeleton organization, multicellular organism development, regulation of intracellular signal transduction, cell communication, cell surface receptor signaling, regulation of programmed cell death etc.), followed by molecular function (cellular macromolecular catabolic processes, kinase activity, phosphotransferase activity, DNA binding, metal ion binding) and cellular components (ribonucleoprotein complexes, bounding membrane of organelles). KEGG pathway analysis revealed multiple enriched pathways including apoptosis, chemokine signaling, cellular, senescence, mitophagy, lysosome, endocytosis, and MAPK (Figure 3b and Table S4). Detailed analysis of selected enriched pathways is presented in the respective sections below.

### 3.3. Expression of Genes Involved in the Innate Immune Response

**Figure 4:**
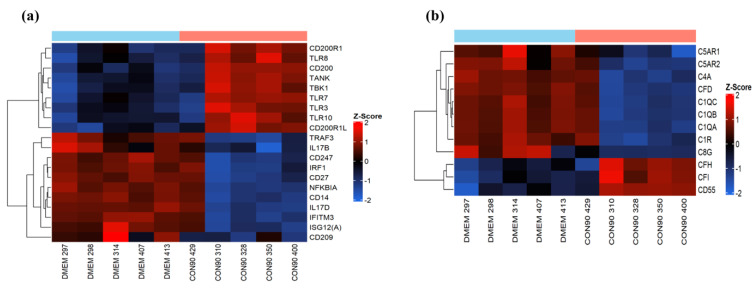
Genes involved in innate immune pathways. Hierarchical clustering analysis of gene expression profile. Each column represents one pig with given treatment and each horizontal line refers to a gene. The associated dendrogram is shown on the left of the heat map. Color legend is on right of the figure. Red indicates genes with increased expression, while blue indicates genes with reduced expression relative to the geometrical means. (**a**) Heatmap showing gene expression profile of innate immune genes; (**b**) heat map showing the profile of differentially expressed genes involved in complement pathways.

The innate immune system is the first line of defense against invading pathogens, with the major influence on the development of strong adaptive immune responses. CON90 is capable of inducing type I IFNs both in vitro and in vivo [27]. In the current study, we did not detect differential expression of type I IFN or interferon stimulated genes (ISG) RNA transcripts, suggesting that the type-I IFN was not induced in ILN of CON-infected pigs at 46 dpi. We observed an upregulation of RNA-sensing molecules including TLR3 (dsRNA), TLR7, and TLR8 (ssRNA) (Figure 4a). On the other hand, no differential expression of signaling molecules downstream of TLR pathways including interferon regulatory factors (IRF) IRF3 and IRF7, was observed (Table S2). In addition, we observed an upregulation of TLR10 mRNA in CON90-infected pigs. While the ligands and signaling pathway involving TLR10 remain poorly understood, it has been demonstrated that TLR10 can act as an anti-inflammatory receptor which can also suppress TLR3-induced IFN-β production [45]. Interestingly, we observed an increased expression of CD200 (OX2) and its receptor CD200R (Figure 4a). CD200R is an inhibitory immune receptor that is expressed on myeloid cells and B- and T-lymphocytes [46], while CD200 is widely expressed on multiple cell types including endothelial cells, neurons, and lymphocytes [47]. CD200/CD200R interaction suppresses cytokine production, and inflammatory responses [48].

The complement system is another crucial component of the innate immunity. It acts as an important link between innate and adaptive immune system. Thus, viruses have developed a mechanism to modulate complement responses by down-regulating activation proteins and up-regulation of regulatory proteins [49]. In the current study, increased expression of regulatory proteins (CFH, CFI, and CD55) was observed from CON90-infected animals (Figure 4b). Conversely, C1q, a classical complement component involved in increasing virus neutralizing ability of antibodies [50] was down-regulated (Figure 4b). Collectively, the data suggest that the CON90 virus suppresses both complement activation and inflammatory responses at the site of persistent infection.

### 3.4. Expression of Genes Involved in the Apoptotic Pathway

**Figure 5:**
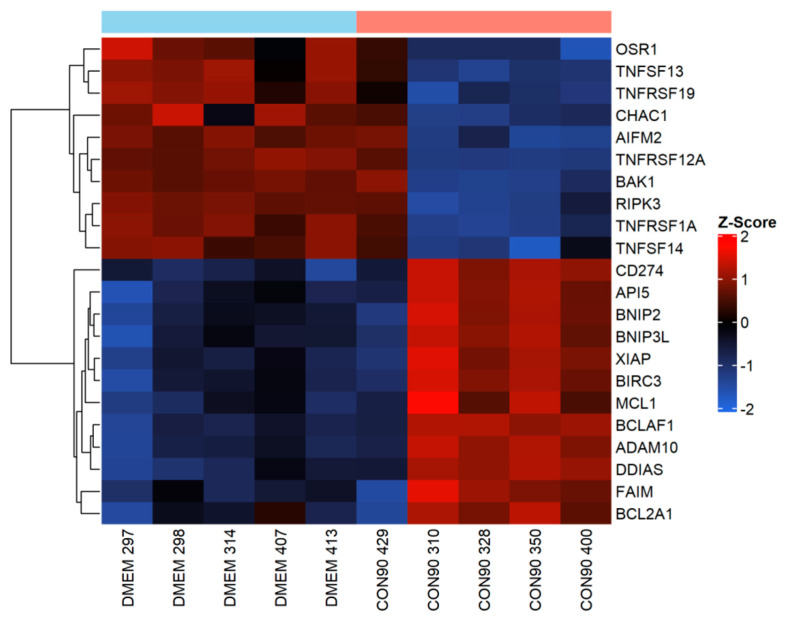
Expression of genes involved in apoptotic regulation in inguinal LN. Heatmap showing gene expression profile of apoptotic genes in ILN tissue from persistently infected and mock-infected animals. This heatmap was generated in the same manner as described in the legend to Figure 4.

Apoptosis is a powerful innate immunity mechanism to curtail viral spread through eliminating virally infected cells. Apoptosis can be triggered by both extrinsic and intrinsic stimuli [51]. It is well documented that PRRSV induces apoptosis in tissues of infected pigs, especially during an acute phase of the infection [15,16,52]. In this study, expression of TNFRSF1A (TNFα receptor 1) gene that contains a death domain [53], was downregulated in the ILN of infected pigs (Figure 5). Similarly, expression of pro-apoptotic genes including AIFM2, CHAC1, and OSR1, was downregulated (Figure 5). On the other hand, expression of BIRC3 and Bfl-1/A1 (BCL2A1), two apoptosis inhibitors that interfere with caspase activation [54], was upregulated. Furthermore, expression of several negative regulators of apoptotic genes including BCL-2-associated killer 1 (BAK1), damage induced apoptosis suppressor (DDIAS), X-linked inhibitor of apoptosis protein (XIAP), MCL1, API, BNIP2, and FAIM was upregulated. Collectively, these results suggest that the pro-apoptotic signaling pathway was suppressed in ILN tissue of pigs persistently infected with CON90.

### 3.5. Expression of Genes Involved in Immune Cell Migration and T-Cell Functions

**Figure 6:**
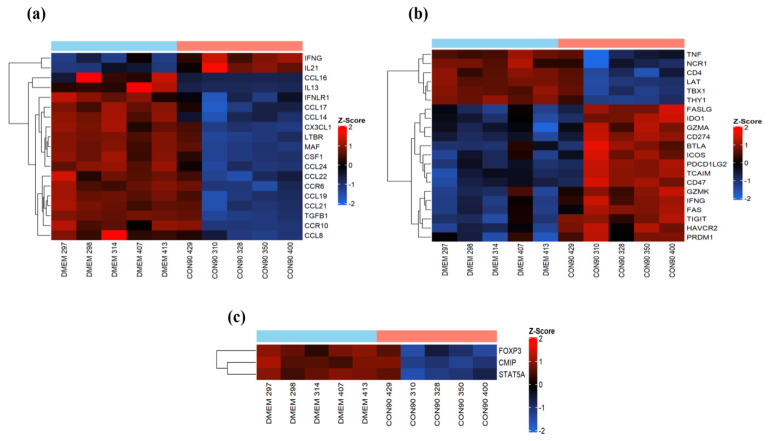
Analysis of cell-mediated immune responses in persistently infected animals. (**a**) Heatmap showing the expression of known cytokines and chemokines and receptors which showed a significant change; (**b**) heatmap showing the genes associated with T-cell development and effector functions; (**c**) heatmap showing the genes involved in T cell-mediated regulatory responses. These heatmaps were generated in the same manner as described in the legend to Figure 4.

Lymphocyte activation occurs in the secondary lymphoid organs. Activated T-cells then migrate to the sites of infection where they exert their effector functions to eliminate infected cells [55]. Cell-adhesion molecules, chemokines, and receptors play a central role in regulating T-cell migration [56]. PRRSV mainly persists in lymphoid tissue of infected pigs [17,18]. In this study, expression of several important chemokine ligands (CCL19, CCL21, CCL24, CCL22, CX3CL1, and CCL14) and chemokine receptors (CCR6 and CCR10) which play an essential role in migration and localization of lymphocytes and antigen-presenting cells (APCs) to the lymphoid tissues was down regulated in ILN of CON90-infected pigs (Figure 6a). On the other hand, expression of CD274 (PD-1), a marker of T-cell exhaustion, was upregulated (Figure 6b). Likewise, expression of inhibitory receptors HAVCR2 (also known as TIM3) and TGIT, which transmit the inhibitory signals for T-cell differentiation and effector activities [57], was upregulated. We also found increased expression of other co-inhibitory molecules (BTLA, FASLG, FAS, and IDO1) that are associated with the regulation of T-cell exhaustion during chronic viral infection (Figure 6b). Together, the results suggest that T-cell migration to ILN, one of the sites of PRRSV persistence, might be affected because of the down regulation of important chemokines and receptors, and that the T-cells in ILN might be exhausted. Interestingly, markers of regulatory T-cells (T_reg_) were downregulated in ILN of infected pigs (Figure 6c), suggesting that T_reg_ might not be present in ILN at 46 dpi.

### 3.6. Frequencies of Virus-Specific T Cells in PMBCs and ILN

**Figure 7:**
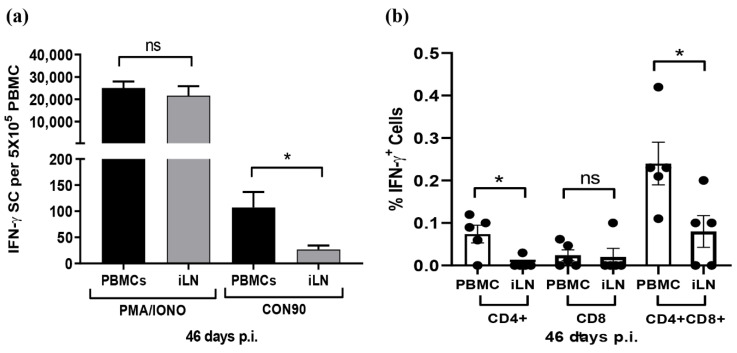
Analysis of IFN-γ secreting cells during persistent PRRSV infection. PBMCs and ILN cells were collected from five pigs that were infected with CON90 at 46 dpi. The cells were stimulated with CON90 as described in the Materials and Methods. For positive control, the cells were stimulated with PMA and ionomycin (PMA/IONO). (**a**) Frequencies of IFN-γ SCs in PBMCs and ILN measured by IFN-γ Elispot. PMA/IONO spots counted as per 5000 cells/well and extrapolated for 5 × 10^5^ cells. (**b**) Percentage subset of T cell secreting IFN-γ as measured by flow cytometric analysis. Dataset represents mean ± S.E.M values calculated from five pigs in each treatment group. * *p* < 0.05.

Since the expression of chemokines and receptors important for T-cell migration was downregulated in ILN, we sought to compare the frequencies of virus-specific T-cells in PBMCs and in ILN using the IFN-γ SC Elispot assay. The number of spots was similar when PBMCs and ILN cells were stimulated with non-specific T-cell activator PHA/Ionomycin. However, the number of spots was significantly lower in ILN than in PBMCs when the cells were stimulated with whole PRRSV antigen (Figure 7a). The results clearly indicate that the frequencies of PRRSV-specific IFN-γ SCs were significantly lower in ILN than in PBMCs. To further elucidate the phenotypes of PRRSV-specific T-cells, we used a multi-color flow cytometric assay to identify subsets of T-cells that secrete IFN-γ in response to PRRSV activation ex vivo. CD4^+^ CD8^+^ DP cells were the major T-cell population secreting IFN-γ, both in PBMCs and ILN. Swine have a significant population of extrathymic CD4^+^ CD8^+^ DP T cells that represent memory T-cells [58]. Similar to the Elispot assay, the flow cytometric assay also indicated that the percentage of T-cell secreting IFN-γ was comparatively lower in ILN than in PBMCs (Figure 7b). Cells collected from DMEM-inoculated pigs did not show any significant T-cell reactivities after ex vivo stimulation with CON90, both in Elispot and in flow cytometry assays (data not shown).

### 3.7. Expression of Genes Involved in the Humoral Immune Response

**Figure 8:**
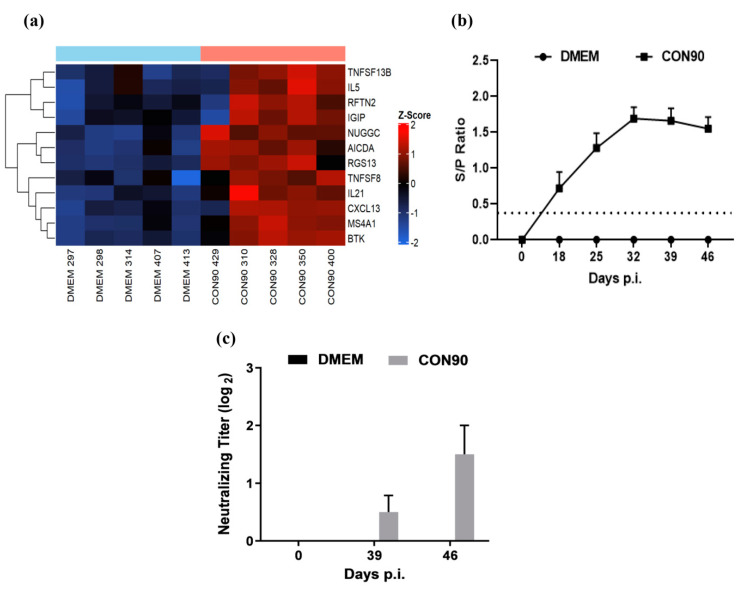
Humoral immune response. (**a**) Heat map of differentially expressed genes associated with the humoral immune development. The heatmap was generated in the same manner as described in the legend to Figure 4. (**b**) PRRSV-specific antibodies were detected in the serum using IDEXX PRRS X3 Antibody test (IDEXX Laboratories, lnc., Westbrook, ME, USA). The horizontal dotted line indicates the cutoff (S/P value, 0.4) of the assay. (**c**) Serum virus neutralizing antibodies measured against CON90 at 39 and 46 days post infection. Dataset represents mean ± S.E.M values calculated from five pigs in each treatment group.

A number of genes associated with Th2, or humoral response, were upregulated in the ILN of CON90-infected pigs (Figure 8a). IL-21, RGS13, and NUGGC are involved in the development of germinal center (GC) and activation of B-cell follicles [59]. TNFSF13B and TNFSF8 are potent activators of B-cell lineage and Ig class switching, respectively [60]. B-cell surface antigens MS4A1 (CD20), activation-induced cytidine deaminase (AICDA) [61], and rafting family member 2 (RFTN2) are associated with B-cell receptor signaling. The upregulated expression of these genes in ILN of infected animals suggested that the humoral immune response to CON90-infection was not affected. To corroborate transcriptome data, we measured both virus-specific antibody levels at various time points post-infection. High levels of non-neutralizing antibodies specific to viral N protein (measured by a commercial ELISA) were detected in all pigs (Figure 8b). However, only minimal levels of virus-neutralizing antibodies (titer 1:2) were detected in the serum of infected pigs at 46 dpi (Figure 8c). Together, both transcriptomic and serological data indicate that B-cell development and antibody production are not affected by CON90-infection. However, low levels of virus-neutralizing antibodies are likely due to the virus ability to escape antibody neutralization (see below).

## 4. Discussion

PRRSV persists in lymphoid tissue of infected pigs for several months [17,18]. The mechanism of PRRSV persistence is not fully understood. We performed genome-wide transcriptome analysis of lymphoid tissue collected from pigs persistently infected with an attenuated PRRSV strain using RNA-seq technology that detects both host and viral RNA. Viral RNA reads were detected in ILN of all five infected pigs. It was reported previously that PRRSV genome mainly exists in dsRNA forms in lymphoid tissues during persistent infection [26]. Since only mRNA (purified by using poly-T oligo-attached magnetic beads) was used for library construction and RNA sequencing, the viral RNA reads detected in this study must be derived from either viral genomic RNA or sub-genomic mRNA, not from dsRNA. The viral RNA reads map throughout the viral genome. However, we are not able to discern whether these reads are derived from genomic or sub-genomic mRNA because we used a short-read RNA sequencing platform. It would be interesting to use long-read RNA sequencing to study the viral transcriptome at different states of infection in pigs [62].

It was reported previously that no significant DEGs were observed in lymphoid tissue of persistently infected animals [26]. In the current study, we identified a large number of DEGs in persistently infected animals (Figure 2b). This might be due to the difference in the experimental setup. In the previous study, pigs were infected with a wild-type PRRSV-1 whereas in this current study, pigs were infected with an attenuated synthetic PRRSV strain [27,63]. Besides, the previous study looked at a small set of selected genes while in this study we look at the genome-wide RNA transcriptome. 

GO terms and KEGG analysis revealed that genes involved in the innate immune (complement and TLR) pathways, apoptosis, cytokine-chemokine signaling, T-cell exhaustion, and humoral responses are highly differentially expressed in PRRSV-infected animals compared to control. The complement system is a constituent of innate immunity that serves by neutralizing cell-free viruses, lysing virus-infected cells, and boosting virus specific responses [64]. It also links the innate and adaptive immune responses, enhances humoral immunity, regulates antibody effector mechanisms, and modulates T-cell function [65]. Many viruses have developed a strategy to evade the complement pathway by recruiting or enhancing the production of host transcription regulatory components. During acute infection (7 dpi), PRRSV significantly represses the expression of complement regulatory components (CD55 and C4BPB) in lung tissue [16]. Early complement activation during acute infection facilitates the release of newly formed virions from infected cells, yet, chronic viral infection is reported to suppress the activity of complement activation proteins and increases the activity of regulatory proteins (Reviewed [49]).

In the current study, we found overexpression of CFH and CFI along with another regulatory factor CD55 (decay-accelerating factor). Overexpression of CFH, a major soluble regulator of the alternative pathway, results in inhibition of C3 and C5 convertase enzymes. CFI suppresses the complement active proteins C3b (opsonin) and C4b via mediating cleavage to their inactive form [66,67]. Regulatory factor CD55 is an inhibitor of C3 convertase which prevents C3b deposition on the cell surface [68]. Apart from that, we also see the downregulation of C1q, which increases neutralizing and hemagglutination inhibition activity of anti-influenza antibodies [50]. Available data from previous studies and the current study suggest that activation of complement pathway during acute infection helps to disseminate virus, while suppression of complement components like C1q, C1r, and C5, and upregulation in regulatory components during persistent infection facilitates the virus to escape from the complement system to maintain persistence in lymphoid tissues.

Most naturally occurring PRRSV strains suppress type I IFN production by inhibiting the activation and nuclear translocation of IRF3/IRF7 and NF-kB [69,70]. Deficiency of IRF3/7 results in severe mortality to infection with West Nile virus (WNV), Chikungunya virus (CHIKV), and Ross River virus infection, and promotes viral persistence in the infected hosts [71,72,73]. It has been reported that PRRSV nsp1β inhibits IRF3 phosphorylation and nuclear translocation; thus, inhibiting IFN production [69,74,75]. Interestingly, the synthetic PRRSV-CON and its attenuated form CON90 were able to induce type I IFNs [27,76]. Contrary to its nature to induce type I IFN, in the current study no upregulation of canonical type I IFN signaling pathway-associated genes was observed. However, expression of RNA sensing molecules including TLR3, TLR7, and TLR8 was upregulated in CON90-infected animals. On the basis of available data, we hypothesize that reduced levels of viral replication and sequestration of viral RNA in infected cells during persistent infection might limit further activation of type I IFN signaling pathway by preventing interaction with cytoplasmic pattern recognition receptors (PRRs).

Apoptosis or programmed cell death is a potential host immune mechanism against virally infected cells to curtail the spread of newly formed viral progenies. Apoptosis is induced by two distinct, yet inter-connected signaling pathways, the extrinsic and intrinsic pathways [51]. In order to successfully establish persistent infection, viruses have developed mechanisms to inhibit apoptosis. For instance, adenovirus (E1B-19K), human cytomegalovirus (UL37), poxviruses (F1L), and myxoma virus (M11L) have an inhibitory effect on proapoptotic proteins Bak/Bax [77]. It appears that PRRSV can also modulate apoptosis. Studies of pulmonary alveolar macrophages (PAM) infected with PRRSV in vitro reveal that the virus stimulates anti-apoptotic pathways early in infection while it induces apoptosis late in infection [78]. On the other hand, the virus induces apoptosis in the tissues of infected animals during acute infection, but the frequencies of apoptotic cells reduced to normal levels observed in control, non-infected pigs from day 20 post-infection [52]. In this study, expression of multiple anti-apoptotic genes including XIAP, Bfl-1/A1 (BCL2A1), and BIRC3 was upregulated while expression of pro-apoptotic genes (AIFM2, CHAC1, and OSR1) was downregulated. XIAP is an endogenous caspase 9 and 3 inhibitor [79]. Bfl-1/A1 (BCL2A1) is a transcriptional target of nuclear factor-kB (NF-kB) that suppresses caspase activation and apoptosis in response to death-inducing stimuli like TNFα [54]. BIRC3 is an interaction partner to TNFRSF1B and inhibits apoptosis by interfering with the caspase activation [53]. The pro-apoptotic BCL-2 family member like BCL-2-associated killer 1 (BAK1), which allows the release of cytochrome C via formation of homo-oligomers and stable insertion into outer mitochondrial membrane was significantly suppressed. Thus, the data suggest that apoptosis was suppressed in the ILN of CON90-infected animals during persistent infection.

Chemokine molecules CCL19 and CCL21 play a crucial role in migration, activation, expansion, and survival of antiviral T-cells. Suppression of the CCR7 and CCL19/CCL21 axis results in dysfunction of T-cells during viral infection [80,81]. CCL19-CCR7 axis plays role in protection against multiple viruses, such as HIV [82], herpes simplex virus (HSV-1) [83], HSV-2, hepatitis C virus [84], and pseudorabies [85]. Acute PRRSV infection increases the expression of chemokines, leading to the infiltration of immune cells toward the sites of infection [16,86]. In this study, expression of both CCL19 and CCL21 was significantly downregulated in CON90-infected lymph node (Figure 6a). Additionally, expression of different chemoattractant molecules (fractalkine/CX3CL1, CCL14, CCL16, CCL17, CCL22, and CCL8) and receptors (CCR6 and CCR10) was also downregulated. It is possible that downregulation of these chemokines and receptors might impair T-cells trafficking to inguinal lymph node, the site of PRRSV persistence. Our transcriptional data are supported by the functional data which demonstrate that the frequencies of IFN-γ SC in ILN were significantly lower than in PBMCs (Figure 7).

Chronic or persistent viral stimulation results in hierarchical loss of effector functions of T-lymphocytes, including proliferation, cytokine production (e.g., IFN-γ and IL-2), and cytolytic responses [57,87,88]. Although the molecular signatures involved in T-cell exhaustion are not completely understood, the overexpression of cell surface inhibitory receptors (e.g., PD-1, CTLA-4, and others) primarily mediates CD8+ T-cell dysfunction [87,88,89]. T-cell exhaustion seems to be a common phenomenon caused by arteriviruses, as T-cell exhaustion was also reported in horses persistently infected with EAV [24]. Recently, it was shown that PRRSV infection alone or co-infection with porcine circovirus type 2 (PCV2) can significantly upregulate surface expression of PD-L1 (CD274) on porcine monocytes-derived dendritic cells (MoDCs) [90]. Increased expression of PD-L1 on the surface of antigen presenting cells (APCs) possibly contributes to the ineffective T-cell responses during the PRRSV infection. In the present study, expression of several markers for T-cells exhaustion such as PD-1 (CD279) and its ligand, CD274 (PD-L1) was upregulated in CON90-infected animals, suggesting that the T-cells in lymph nodes of persistently infected animals might be exhausted (Figure 6b). However, additional studies (e.g., T-cell cytotoxicity and cytokine production) are required to fully assess the functionality of T-cells during PRRSV persistence.

Germinal centers (GCs) are the specialized locations in the secondary lymphoid tissues where B-cell maturation, differentiation, somatic hypermutation, and class switching of isotypes take place [91]. In the current study, the GC development-associated genes, which includes IL-21 produced by follicular helper T-cells [61], the regulator of G protein signaling (RGS13) [92], and GC-associated nuclear GTPase (NUGGC) [59], were upregulated in the lymph node of CON90-infected animals. Thus, PRRSV infection does not seem to impair GC formation and B-cell activation. This is supported by the fact that all five CON90-infected pigs developed a robust antibody response, as measured by the IDEXX ELISA (Figure 8b). However, only minimal titers of virus-neutralizing antibodies were detected at 46 dpi. Perhaps, PRRSV does not suppress the humoral immune response. Instead, the virus evades from antibody-mediated neutralization through different mechanisms such as glycan shielding and decoy epitopes [93,94,95].

## 5. Conclusions

In summary, we identified a robust host transcriptional response in the inguinal lymphoid tissue of pigs persistently infected with the attenuated PRRSV strain CON90. Genes involved in the innate immune responses are downregulated. Similarly, chemokines and receptors associated with T-cell homing to the lymphoid tissue are downregulated. This might lead to the lower frequencies of virus-specific T-cells in lymphoid tissue than in peripheral blood. Additionally, the genes involved in the anti-apoptotic pathways are upregulated. Collectively, the data suggest that PRRSV can create a pro-survival microenvironment at the lymphoid tissue which allows the virus to persist for an extended period.

## Supplementary Materials

The following are available online at https://www.mdpi.com/1999-4915/12/8/817/s1. Figure S1. Representative flow cytometry gating strategy used to identify IFN-γ secreting CD4α^+^, CD8α^+^, and CD4α^+^CD8α^+^ (DP) T cells from PBMCs and ILN. Table S1. Summary of sequencing quality control and mapping data of samples. Table S2. DEGs identified in group DMEM vs. CON90. Table S3. Detail information of GO enrichment of DEGs in DMEM vs. CON9. Table S4 Detail information of KEGG pathway enrichment of DEGs in DMEM vs. CON90.
