# Supplementary material for: Host Transcriptional Response to Persistent Infection with a Live-Attenuated Porcine Reproductive and Respiratory Syndrome Virus Strain
Source: Viruses. 2020 Jul 28;12(8):817. doi: 10.3390/v12080817 (PMC7474429; doi:10.3390/v12080817)
Supplement: Supplementary file 1 [file viruses-12-00817-s001.zip › Supplementary/Table S1.docx]

| **Sample name** | **Raw reads** | **Clean reads** | **Raw bases** | **Clean bases** | **GC content (%)** | **Total mapped** | **Multiple mapped** | **Uniquely mapped** |
| --- | --- | --- | --- | --- | --- | --- | --- | --- |
| DMEM 297 | 23993578 | 23911096 | 3.6G | 3.6G | 59.75 | 6564049 | 6360625 | 203424 |
| DMEM 298 | 24638558 | 24568732 | 3.7G | 3.7G | 52 | 5330848 | 5202980 | 127868 |
| DMEM 314 | 20602920 | 20564058 | 3.1G | 3.1G | 56 | 5772545 | 5485918 | 286627 |
| DMEM 407 | 26412820 | 26312124 | 4.0G | 3.9G | 54.25 | 7804300 | 7482504 | 321796 |
| DMEM 413 | 20308824 | 20250074 | 3.0G | 3.0G | 55.5 | 5637488 | 5447107 | 190381 |
| CON90 310 | 51895766 | 51715936 | 7.8G | 7.8G | 39 | 15214713 | 15105283 | 109430 |
| CON90 328 | 45425268 | 45355796 | 6.8G | 6.8G | 41 | 13214350 | 13118667 | 95683 |
| CON90 350 | 48132518 | 47961302 | 7.2G | 7.2G | 40 | 13932711 | 13828835 | 103876 |
| CON90 400 | 40355686 | 40295502 | 6.1G | 6.0G | 41.5 | 11446707 | 11363463 | 83244 |
| CON90 429 | 22810860 | 22770396 | 3.4G | 3.4G | 56.75 | 6337935 | 6124624 | 213311 |

**Table. 1**. Summary of sequencing quality control and mapping data of samples
