# Supplementary material for: Host Transcriptional Response to Persistent Infection with a Live-Attenuated Porcine Reproductive and Respiratory Syndrome Virus Strain
Source: Viruses. 2020 Jul 28;12(8):817. doi: 10.3390/v12080817 (PMC7474429; doi:10.3390/v12080817)
Supplement: Supplementary file 1 [file viruses-12-00817-s001.zip › Supplementary/Figure S1.docx]

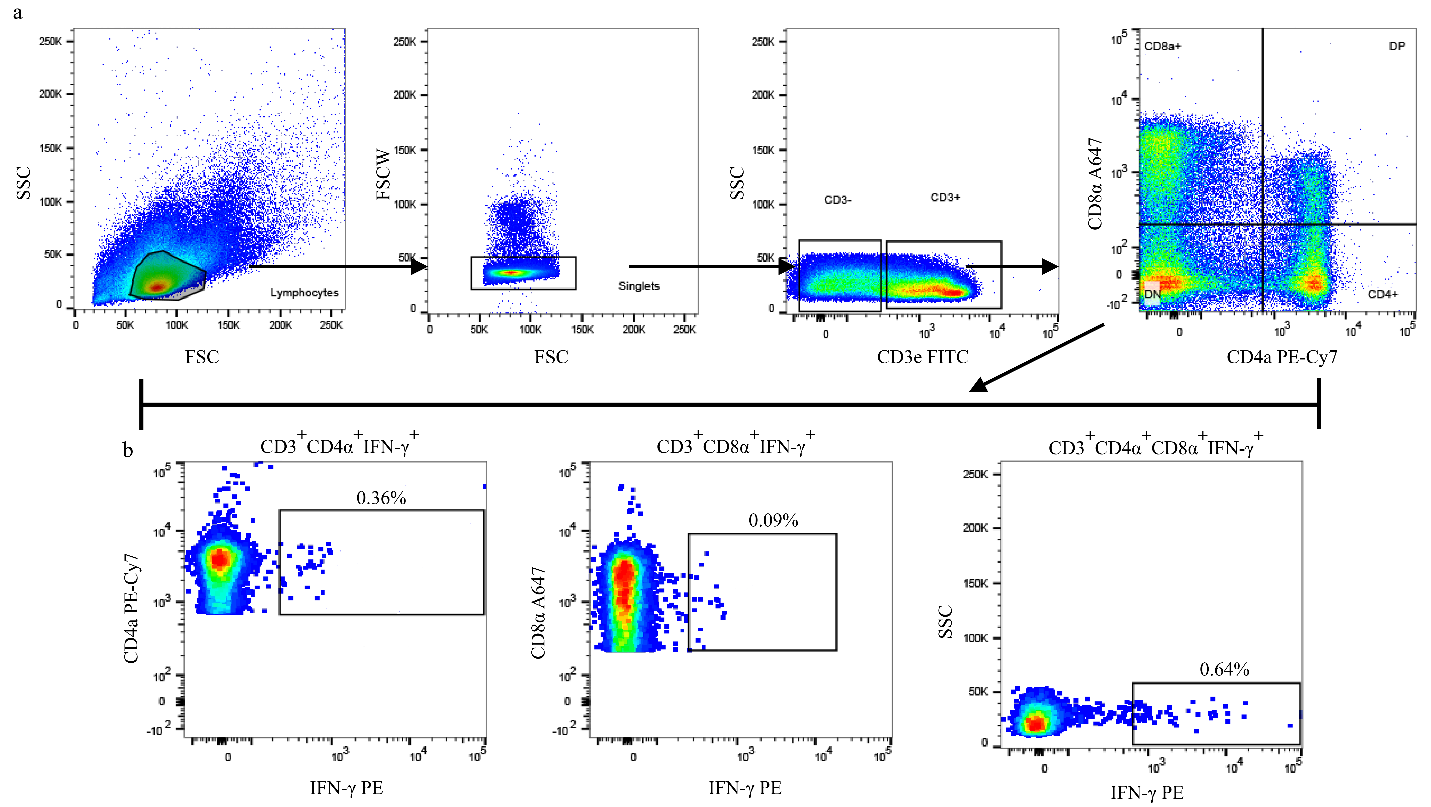


**Figure S1**. Representative flow cytometry gating strategy used to identify IFN-ϒ secreting CD4α^+^, CD8α^+^, and CD4α^+^CD8α^+^ (DP) T cells from PBMCs and ILN. Single cell suspension of PBMCs and ILN cells in vitro stimulated with CON90 or cRPMI and IFN-ϒ assessed by flow cytometry. Both cell types were stained with CD3, CD4α, CD8α and IFN-ϒ monoclonal antibodies (MAb). (a) Both PBMCs and ILN cells with a low side scatter (SSC) profile were selected using forward (FSC) and side scatter (SSC) detector. Subsequently, singlet cell population further divided into CD3- and CD3+ fractions and T-cell population were defined as CD3^+^CD4α^+^, CD3^+^CD8α^+^, and CD3^+^CD4α^+^CD8α^+^ (DP). (b) Representative data of the pig CON90 400 showing expression of intracytoplasmic IFN-ϒ in each T-cell population.
